# Wisconsin’s Screening Algorithm for the Identification of Newborns with Congenital Adrenal Hyperplasia

**DOI:** 10.3390/ijns5030033

**Published:** 2019-09-06

**Authors:** Eric R. Bialk, Michael R. Lasarev, Patrice K. Held

**Affiliations:** 1 Wisconsin State Laboratory of Hygiene, University of Wisconsin School of Medicine and Public Health, Madison, WI 53706, USA; 2 Department of Biostatistics and Medical Informatics, University of Wisconsin School of Medicine and Public Health, Madison, WI 53726, USA; 3 Department of Pediatrics, University of Wisconsin School of Medicine and Public Health, Madison, WI 53792-4108, USA

**Keywords:** newborn screening, congenital adrenal hyperplasia, second-tier testing, liquid chromatography–tandem mass spectrometry

## Abstract

Newborn screening for congenital adrenal hyperplasia (CAH) has one of the highest false positive rates of any of the diseases on the Wisconsin panel. This is largely due to the first-tier immune assay cross-reactivity and physiological changes in the concentration of 17-hydroxyprogesterone during the first few days of life. To improve screening for CAH, Wisconsin developed a second-tier assay to quantify four different steroids (17-hydroxyprogesterone, 21-deoxycortisol, androstenedione, and cortisol) by liquid chromatography–tandem mass spectrometry (LC–MSMS) in dried blood spots. From validation studies which included the testing of confirmed CAH patients, Wisconsin established its own reporting algorithm that incorporates steroid concentrations as well as two different ratios—the birth weight and the collection time—to identify babies at risk for CAH. Using the newly developed method and algorithm, the false positive rate for the CAH screening was reduced by 95%. Patients with both classical forms of CAH, salt-wasting and simple virilizing, were identified. This study replicates and expands upon previous work to develop a second-tier LC–MSMS steroid profiling screening assay for CAH. The validation and prospective study results provide evidence for an extensive reporting algorithm that incorporates multiple steroids, birth weight, and collection times.

## 1. Introduction

Congenital Adrenal Hyperplasia (CAH) is a group of autosomal recessive disorders caused by enzymatic defects within the biosynthetic pathways of mineralocorticoids, glucocorticoids, and sex hormones. The most common defect, accounting for greater than 90% of CAH cases, is due to 21-hydroxylase (21-OH) deficiency, and has an estimated prevalence in the United States of 1 in 16,000 to 1 in 20,000 [1,2,3]. There are three presentations of 21-OH deficiency that range in severity: classical salt-wasting (SW), classical simple virilizing (SV), and non-classical. Patients with SW-CAH may experience a life-threatening adrenal crisis within the first week of life, leading to hyponatremia, dehydration, alkalosis, and shock. Early treatment with glucocorticoid and mineralocorticoid replacement is effective and lifesaving [1,2,3].

Newborn screening for 21-OH deficiency was first introduced in 1977 [4], with the objective to identify patients with the severe salt-wasting form (SW-CAH) of the disease to provide therapeutic intervention and reduce mortality [5,6]. Wisconsin added CAH to its newborn screening panel of diseases in 1983. All states utilize a first-tier immunoassay, measuring 17-hydroxyprogesterone (17-OHP) in dried blood spot specimens [7]. Although screening for CAH using 17-OHP is effective at identifying severely affected patients at risk for salt-wasting crisis, there is a high false positive rate associated with the assay [7]. Assay limitations, such as cross-reactivity with other steroid sulfatides [8], and physiological differences between newborns particularly within the premature infant population, contribute to the high false positive rate [9,10]. The use of alternative antibodies within the assay and modifications to cutoff values based on birth weight and/or gestational age has not substantially reduced the burden of mis-identification [11,12,13]. At present, 9 states mandate a second screen on all newborns at 9–14 days of life [14]. This additional screen can impact the positive predictive value of CAH testing, however, the logistical and financial burden is large. 

In recent years, newborn screening programs have implemented second-tier testing to reduce false positive rates. This second-tier test is typically performed on the original specimen, when the first-tier screening result—elevated 17-OHP—is abnormal. In 2004, Lacey et al. published the first report of a second-tier method for CAH [15]. This method extracted cortisol (F), 17-OHP, and androstenedione (4-A) from the dried blood spot specimen using diethyl ether, followed by separation, identification, and quantification of the three steroids using liquid chromatography–tandem mass spectrometry (LC–MSMS). Modifications to this original method, eliminating the diethyl ether extraction, were published by other state newborn screening programs; however, states continued to only quantify cortisol, 17-OHP, and androstenedione [16,17]. Research groups later demonstrated the feasibility of analyzing a larger panel of steroids in dried blood spots using LC–MSMS with up to 9 analytes, including 11-deoxycortisol (11) and 21-deoxycortisol (21) [18,19]. Additional reports have highlighted the utility of 21-deoxycortisol, measured in serum, as a marker of 21-OH deficiency [20,21].

In 2007, Janzen et al. published an original method validation and prospective study which incorporated analysis of 21-deoxycortisol in dried blood spots, along with the ratio of (17-OHP + 21)/F [22]. The article highlighted differences in steroid concentrations across a range of gestational ages. While comprehensive, the article did not consider the collection timing of the specimen, nor did it provide a testing algorithm. A more recent pilot study for CAH screening performed in Turkey used only the ratio of (17-OHP + 21)/F, stratifying by gestational age and birth weight, to identify cases of CAH. Timing of blood spot collection within this study was considerably later (3–5 days) than practices of state newborn screening programs [23]. To date, a prospective screening study for CAH—including second-tier LC–MSMS analysis of a steroid panel and multiple associated ratios—has not been reported. Likewise, a comprehensive screening algorithm, incorporating first- and second-tier analyses and stratification by birth weight and timing of collection, has not been proposed. 

This study adds to the literature by outlining the method validation for four different steroids measured by LC–MSMS. Details on the development of an algorithm, which incorporates first-tier testing and second-tier analysis of multiple steroids and associated ratios, stratified by birth weight and the timing of specimen collection, are provided. Lastly, this study summarizes the year-long implementation of the second-tier test and its influence on positive predictive value and false positive screening rates for CAH within the Wisconsin population.

## 2. Materials and Methods

### 2.1. Dried Blood Specimens

A protocol (2019-0118) for the use of residual dried blood spot (DBS) newborn screening (NBS) specimens was approved by the Health Sciences Institutional Review Board (IRB) at the University of Wisconsin (3/28/2019). For the assay validation, DBS specimens from 18 patients with CAH were obtained from newborn screening laboratories and were de-identified according to the IRB-approved protocol. Residual, known false positives specimens (*n* = 144), obtained through the Wisconsin NBS program, were also used for the validation. The true and false positive specimens were stored at −20 °C prior to analysis. For the prospective study, all dried blood spot specimens were collected between 0 and 168 h after birth and were stored at 4 °C after routine NBS was completed.

### 2.2. Reagents and Standards

Standards for cortisol (F) and 17-hydroxyprogesterone (17-OHP) were purchased from Sigma (St. Louis, MO, USA). The standard for 21-deoxycortisol (21) was purchased from ChemCruz (Dallas, TX, USA), and the standard for 4-androstene-3,17-dione (4-A) was obtained from Cambridge Isotopes (Tewksbury, MA, USA). The isotopically labeled internal standards D-7, 4-androstene-3,17-dione (2,2,4,6,6,16,16), D-8, 21-deoxycortisol (2,2,4,6,6,21,21,21), D-8, 17-hydroxyprogesterone (2,2,4,6,6,21,21,21), and D-4, cortisol (9,11,12,12) were purchased from CDN isotopes (Pointe-Claire, Greater Montreal, QC, Canada). Acetonitrile and methanol HPLC–MS grade solvents were purchased from J.T. Baker through Fisher Scientific (Pittsburgh, PA, USA). Grade 903 filter paper for DBS preparation was purchased from Whatman GmbH (Dassel, Germany). Sera Con II charcoal filtered de-lipidated plasma was acquired from SeraCare Life Sciences (Milford, MA, USA). Packed red blood cells (RBC) from *O*-positive donor and plasma were purchased from the American Red Cross (St. Louis, MO, USA). Plasma and washed RBC were combined in 50/50 *v*/*v* (to achieve a 50% hematocrit) to make whole blood.

### 2.3. Quality Control and Calibrators 

Three different sets of quality control materials for the four steroids at varying concentrations were prepared. Whole blood was enriched with 15 ng/mL or 150 ng/mL of 17-OHP, 11, 21, and 4-A to prepare the low- and high-quality control materials respectively. The normal control was not inoculated with 17-OHP, 21, or 4-A. Cortisol was inversely spiked into the normal, low- and high-quality control materials at concentrations of 80, 15 and 5 ng/mL, respectively, to mimic patient profiles with high 17-OHP and low cortisol concentrations.

Calibrators were prepared with equimolar enrichment of each steroid in the following levels: 0, 2.5, 5.0, 10, 50, 100 and 250 ng/mL. These calibrators were also used to assess the method’s linearity and limit of quantification.

The unenriched and enriched whole blood pools used in creation of the quality control materials and calibrators were dispensed onto filter paper, dried overnight under ambient conditions, and stored at –20 °C in zip-closure plastic bags containing desiccant packets.

### 2.4. First-Tier Screening Assay for the Quantification of 17-Hydroxyprogesterone

**Figure 1:**
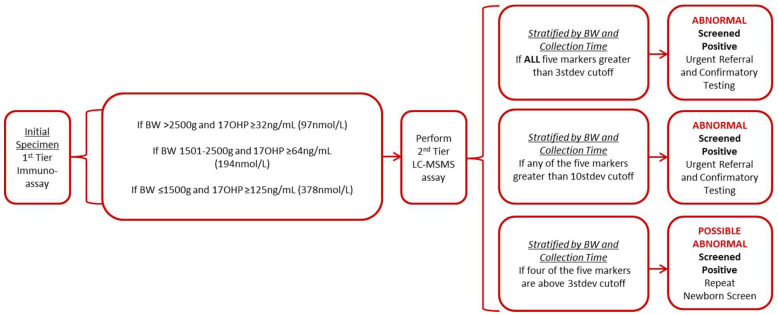
Newborn screening algorithm for congenital adrenal hyperplasia. Figure 1 provides the screening algorithm used to detect cases of congenital adrenal hyperplasia which includes both the first-tier assay and the second-tier assay, stratified by birth weight and collection time, and the corresponding interpretation guidelines. The five markers used for interpretation are 17OHP, 4-A, 21, [(17-OHP + 4-A)/F] and [(17-OHP+21)/F]. BW = birth weight.

As part of routine screening within Wisconsin, whole blood samples obtained from newborns between 0 and 168 h after birth were collected on Whatman GmbH grade 903 filter paper, dried, and sent to the Wisconsin NBS laboratory. 17-OHP was measured on all specimens using a time-resolved fluoroimmunoassay according to the manufacturer’s specifications (GSP; PerkinElmerLife and Analytical Sciences; Shelton, CT, USA). The results of this first-tier CAH screening were interpreted according to the birth weight of the newborn (Figure 1). Any specimen with a 17-OHP value greater than the cutoff was analyzed by second-tier screening assay.

### 2.5. Second-Tier Screening Assay for Quantification of Five Steroids 

A 3.2 mm (⅛”) punch of the DBS specimen was placed into a 96-well, round-bottom polypropylene plate. The steroids (F, 17-OHP, 21, 4-A) were extracted from the punch using 175 µL of an 80:20 acetonitrile/water solution containing 3 ng/mL of each internal standard, followed by shaking at 50 °C for 60 min. The extraction solution was transferred to a clean 96-well plate and dried under nitrogen gas for 35–40 min at a temperature of 40 °C. The dried well was reconstituted using 50 µL of 40:60 methanol/water solution with 0.3% formic acid and then shaken for 10 min at room temperature, prior to analysis.

The steroids were separated by liquid chromatography using a Phenomenex Kinetex (Torrance, CA, USA) 5 µm C18 110A 50 × 2.1 mm column and analyzed using an AB Sciex API 4500 tandem mass spectrometer with a TurboV electrospray ionization source. The API 4500 was operated in positive-ionization mode with the ion spray voltage set to 1500 V, declustering potential 70 V, excitation potential of 10 V, and a collision cell exit potential of 13 V. Additional steroid dependent parameters are in Table 1. Two mobile phases of water with 0.1% formic acid and 10 µM ammonium formate (mobile phase A) and 100% methanol with 0.1% formic acid and 10 µM ammonium formate (mobile phase B) were used with the injection profile (Table 2).

Adequate baseline separation of the four steroids, the column wash and re-equilibration was achieved with a total run time of nine minutes. The injected sample volume was 20 µL. The steroids and associated internal standard were analyzed using the multiple reaction monitoring (MRM) pairs in Table 1. Steroid identities were confirmed by their retention time as compared to the labeled internal standard and by the identifying transitions (I). The concentrations were determined using the quantifying transitions (Q) and a linear regression curve with no weighting and forced through zero. All data acquisition and processing were performed using Analyst 1.6.2 software (AB Sciex, Redwood City, MA, USA).

### 2.6. Analysis 

The analytical method described above extracts, identifies, and quantifies four steroids. The described assay validation results below highlight these four steroids (17-OHP, 21, 4-A, and F). To identify newborns with congenital adrenal hyperplasia due to 21-OH deficiency, the following three steroids (17-OHP, 21, and 4-A) and two ratios [(17-OHP + 21)/F and (17-OHP + 4A)/F] were used for interpretation. 

For the population assessment, the four steroid values were log transformed to improve symmetry of the data and stabilize variance across the range of birth weight. Statistical analysis of the data was performed using non-parametric analysis of variance (ANOVA; Kruskal–Wallis test) and Mann–Whitney tests for follow-up pairwise comparisons. Significance was defined as *p* < 0.0001 and all tests are two-sided. 

## 3. Results

### 3.1. Method Validation

A complete clinical assay laboratory validation was performed for four steroids quantified within the second-tier assay (F, 17-OHP, 21, and 4-A). Assay precision was assessed using the two quality control specimens and the unenriched specimen analyzed in six replicates on seven different days. The intra- and inter-assay precision was less than 11% and 15% respectively for all four steroids at varying concentrations exceeding the limit of detection (determined to be 1.0 ng/mL with a 3:1 signal to noise). The limit of quantification was assessed at 2.5 ng/mL for each steroid, with a CV of 26% or less. Linearity ranged from 0 to 500 ng/mL with slopes averaging between 0.83 and 1.15 and a coefficient of determination (*R*^2^) of >0.9970. The recovery of each steroid was within ±15% of expected concentration across the linear range.

### 3.2. Normal Distribution

Routine NBS was performed on 783 specimens, collected between 0 to 167 h (average 24.6 h, median 24 h) after birth. The birth weights for these patient specimens ranged from 300 to 4810 g: 27% with a birth weight less than or equal to 1500 g; 28% with a birth weight between 1501 and 2500 g; and 45% with birth weight >2500 g. The 17-OHP values as measured by the first-tier immunoassay were within the normal range as determined by the birth weight adjusted cutoff (see Figure 1 for birth weight adjusted algorithm). All specimens were analyzed by the second-tier LC–MSMS test to assess the normal population distribution for the four steroids (17-OHP, F, 21, 4-A).

**Figure 2:**
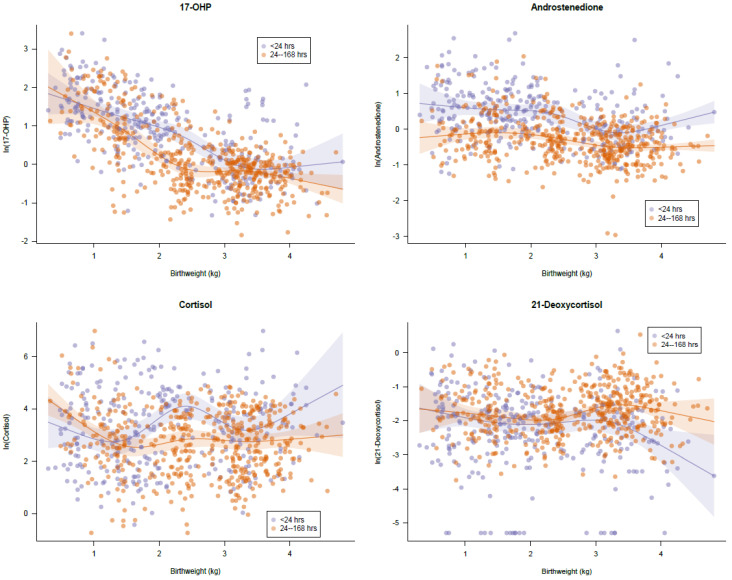
Normal distribution profiles. Figure 2 plots the log-transformed value for each steroid (17OHP, 4-A, F, 21) against the birth weight of the newborn. Dots in orange represent specimens collected prior to 24 h of life. Dots in blue represent specimens collected at 24 h of life or greater.

For each steroid (17OHP, F, 4-A, 21), the data was plotted and a linear regression of the log transformed value against a 5-knot restricted cubic spline for birth weight was fit, with the model stratified according to age at sample collection (before 24 h versus 24–168 h) (Figure 2). 17-hydroxyprogesterone was inversely related to the birth weight of the newborn. This has been shown with previous studies. Additionally, the 17OHP values stratified into three groups based upon the birth weight (≤1500 g, 1501–2500 g and >2500 g) were statistically different (*p*-value <0.0001) as assessed by the one-way ANOVA Kruskal–Wallis test. Androstenedione also declined in concentration with increasing birth weight and was statistically different between each of the three birth weight groups (*p*-values <0.0001). Cortisol and 21-deoxycortisol were not statistically related to birth weight (*p*-values of 0.3217 and 0.0012, respectively).

Steroid concentrations were also assessed by the timing of collection, either before 24 h, or at 24 h or greater; 61% of specimens were collected at 24 h or greater; and 39% of specimens were collected at less than 24 h. The 17-OHP, 4-A, F, and 21 concentrations in specimens collected before 24 h as compared to 24 h or greater were all statistically different (*p*-value <0.0001) as assessed by the Mann–Whitney test. Further stratification by timing of collection and birth weight, using the weight categories listed above, showed statistical differences between groupings for 17-OHP and 4-A (Mann–Whitney *p*-values <0.0001). The Mann–Whitney tests performed on cortisol and 21-deoxycortisol were not consistently significant among the groups (stratified by birth weight and collection time).

The normal range of values for 17-OHP and androstenedione are summarized in Table 3 along with the median, mean, three standard deviation (3stdev) value, and the 99th percentile (0.99). Based upon the analysis above, these two steroids were stratified by both birth weight and collection time. Overall, 21-deoxycortisol was not statistically different when stratified and therefore the data was grouped together using all 783 samples. For the two ratios projected to have utility in the interpretation of second-tier results (17-OHP + 4-A)/F and (17-OHP + 21)/F, the groups were stratified by birth weight, but not by timing of collection, because the Mann–Whitney tests were not significantly different (data not provided). Projected cutoffs based upon the distribution of values across various groups were suggested to be at 3 standard deviations, which correlate similarly to the 99th percentile.

### 3.3. Retrospective Analysis 

#### 3.3.1. Confirmed Cases and False Positives

Second-tier analysis was performed retrospectively on residual specimens from 18 confirmed CAH patients with varying forms of the disease. (See Table 4) In 16 of the 18 patient specimens, the 17-OHP, 4-A, and 21 values and two ratios were above the 3stdev cutoff given the reported birth weight. All of these patients had a classical form of CAH, either salt wasting or simple virilizing. Specimen collection for one patient (#16) was performed at 2 h after birth and only 21, [(17-OHP + 4-A)/F] and [(17-OHP + 21)/F] were elevated; however, the ratios were greater than 10 standard deviations above the mean. In another specimen, collected from a patient (#12) known to have non-classical CAH, only 17-OHP and 21 were elevated; however, the 21 concentration was greater than 10 standard deviations from the mean. All 18 confirmed patients had a birth weight greater than or equal to 2500 grams and specimen collection between 24 to 168 h with the exception of patient 16, whose birth weight was less than 1500 g and specimen collection prior to 24 h. 

The second-tier assay was performed on 144 residual false positive CAH specimens (specimens from patients with abnormal first-tier results but unaffected with disease). Within the 144 specimens, 9 (6.3%) were collected prior to 24 h and 135 (93.7%) were collected at 24 h or later. The birth weight in this population ranged from 403 to 4060 g; 19.4% had a birth weight ≤1500 g, 13.9% were between 1501 and 2500 g, and 66.7% were greater than 2500 g. None of the false positive specimens had all five markers (three steroids and two ratios) greater than 3stdev above the mean, nor were any of the individual markers greater than 10 standard deviations. However, 7 out of 144 specimens (~5%) had abnormal results, greater than 3stdev, for four of the five markers. The remaining 137 specimens had three or fewer markers above the 3stdev cutoff.

#### 3.3.2. Wisconsin Algorithm

An interpretation algorithm was proposed after analysis of the 18 specimens from confirmed cases, 144 false positive specimens, and 783 presumptively normal specimens. All newborns will continue to receive the routine first-tier immune assay to measure the 17-OHP concentration. If the 17-OHP value is determined to be abnormal, depending upon the birth weight adjusted cutoff, the specimen will be referred for second-tier testing. The second-tier test will measure four different steroid concentrations and two ratios. Specimens with 17-OHP, 4-A, 21, and the two ratios [(17-OHP + 4-A)/F and (17-OHP + 21)/F] above three standard deviations will be reported as abnormal (screened positive). Patients will be immediately referred for endocrine consultation and confirmatory testing. Likewise, any sample with any of the five markers (three steroids or two ratios) above 10 standard deviations will also be referred for immediate endocrine consultation and confirmatory testing. To allow for a conservative interpretation and to limit the possibility of false negative results, specimens with four of the five markers above the three standard deviation cutoffs would result in a request for repeat newborn screen See the algorithm in Figure 1. 

As per the Wisconsin protocol for babies in special care units, a routine repeat screen at 14 days of life will continue to be performed on all newborns with a birth weight <2200 g, regardless of the initial CAH result. These repeat specimens will be tested and evaluated using the same algorithm, with the exception of the first-tier 17-OHP cutoff. The repeat specimens will have a more stringent first-tier 17-OHP cutoff, dropping from initial birth weight cutoff to the next lower cutoff (i.e., for a 1100 g baby with an initial cutoff of ≥125 ng/mL for 17-OHP, the cutoff applied to the repeat specimen will be 64 ng/mL for 17-OHP).

### 3.4. Prospective Study Using the Proposed Wisconsin Algorithm

**Figure 3:**
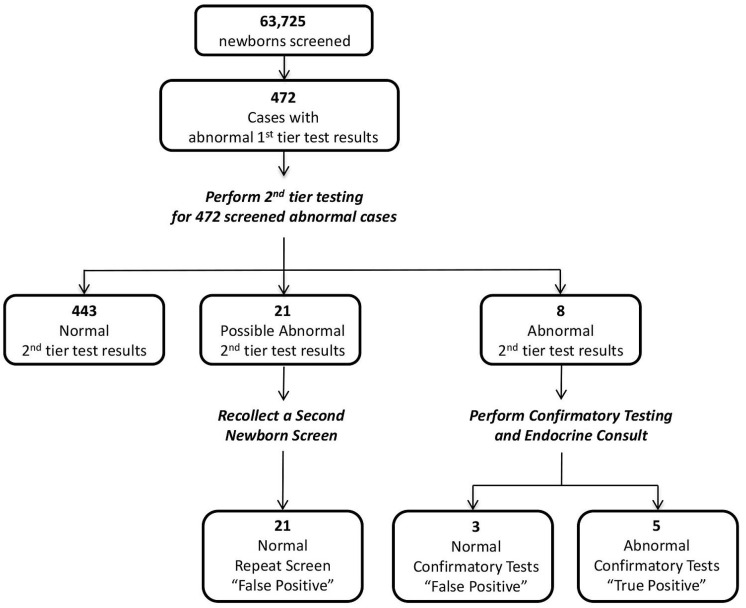
Summary of prospective study. Figure 3 summarizes data from the prospective study. Of the 63,725 newborn screened, there were 472 cases flagged for second-tier testing. Second-tier test results were abnormal for 29 cases. Five of the 29 cases were confirmed to have CAH.

A one-year prospective study incorporating second-tier testing for CAH was implemented into routine newborn screening using the algorithm in Figure 1. The results are summarized below in Figure 3. A total of 63,725 babies were screened during the one-year prospective study, with the average time of collection at 24 h. All first-tier testing was performed within 12 h after receipt of the specimen. Four-hundred and seventy-two babies (0.7% of the total) had an elevated 17-OHP value measured by first-tier analysis above the birth-weight-based cutoff; 66% had a birth weight greater than 2500 g; 18% with birth weight between 1501–2500 g; and 16% has a birth weight ≤1500 g. The second-tier test was performed on the initial screening specimen within 48 h after receiving the abnormal first-tier results. From this group of 472 babies, 29 (6%) were determined to have screened positive second-tier results. The total screening time for all 29 babies, who received both first and second-tier testing, was less than 72 h. Eight of the 29 specimens had elevations of all five markers, and the other 21 specimens had elevations of four of the five markers. The eight newborns with abnormal, screened positive, results were referred for confirmatory testing and endocrine evaluation. Five of the eight babies were confirmed to have classical CAH, either SW or SV, as reported by the endocrinologist or primary care physician. The results of the second-tier testing for the five confirmed babies are highlighted in Table 4 (specimen numbers 19 through 23). The other three babies were determined to be false positives, not affected with CAH. A repeat newborn screen was collected for the 21 newborns with elevations of four markers after second-tier analysis. The second repeat specimens all had normal 17-OHP values as measured by the first-tier test and therefore second-tier testing was not performed. These newborns were considered to be false positives for CAH. To our knowledge, no true cases of CAH were missed by newborn screening (false negatives) during the prospective study.

A calculation of the false positive rates within the prospective study (false positives/total screened) was determined to be 0.75% following first-tier testing, decreasing to 0.04% after second-tier testing and resulting in a greater than 95% reduction in false positive cases. The calculated positive predictive value [true positives/(true positives + false positives)] before and after second-tier testing was also dramatically improved, from 1% to 17%.

## 4. Discussion

The introduction of newborn screening for CAH has profoundly impacted the well-being of affected individuals (approximately 3 to 6 newborns each year in Wisconsin) by reducing the morbidity and the risk of neonatal death due to salt-wasting crisis. However, the current first-tier screening protocol has a high false-positive rate (approximately 0.75% in Wisconsin), leading to unnecessary testing and clinical evaluations, along with parental anxiety and stress [24,25]. The causes of the high false positive rate are many and varied, including limitations of the first-tier assay and physiological factors. The successful implementation of a LC–MSMS second-tier assay for quantification of 17-OHP and other steroids has occurred in several laboratories, with reports of increased assay specificity and positive predictive value, along with a reduction of false positive results by greater than 90% [26,27].

The success of the second-tier test for CAH has been tempered recently with a concern for false-negative results. Previous reports of missed cases have largely been limited to the milder forms of CAH missed by first-tier analysis [28,29,30]. However, a recent article from the Minnesota newborn screening program questioned the sensitivity of the second-tier tests. In a 10-year review, Minnesota reported 15 false negative cases of CAH; patients with either the SV or SW form. Of the 15 missed cases, 4 cases had a normal 17-OHP first-tier test results, while 11 cases had abnormal first-tier results but normal second-tier test results [31]. Missed cases after second-tier analysis raises concerns for the validity of the algorithm. It is possible that additional steroids and ratios, along with factors such as birth weight, gestational age, or the timing of collection need to be considered. Given that false negative cases are often not reported to NBS programs, it is likely that other programs using a simplified second-tier testing algorithm may also miss cases.

This study describes the Wisconsin experience with the validation and implementation of the second-tier LC–MSMS test, including the development of an algorithm for the interpretation of results. Wisconsin chose to analyze four different steroids (17-OHP, 4-A, 21, and F). Assay performance metrics, including precision, accuracy, and linearity, were all consistent with previously reported studies. As part of the assay validation, a complete analysis of the steroid concentrations within the normal population was performed. Consistent with literature reports, steroid concentrations change over time and are also impacted by physiological differences between a premature, low-birth-weight baby compared to a full-term baby [32]. Therefore, Wisconsin stratified the normal population range by both the timing of collection and the birth weight of the newborn. Birth weight instead of gestational age was used as a marker of prematurity, because gestational age is often not collected by state programs or may be inaccurate. Likewise, stratification by collection times, particularly before or after 24 h, is important to consider as state practices continue to push for earlier collection times [33].

From the assessment of the normal population, along with false positive and confirmed cases, an interpretation algorithm including multiple steroids (17-OHP, 21, 4-A) and two additional ratios, stratified by birth weight and specimen collection timing, was proposed. Sixteen of the confirmed cases of classical CAH (SV or SW) had all five markers elevated above 3 standard deviations from the mean. There was, however, a dramatic range in steroid concentration within the confirmed cases (17-OHP ranged from 6.14 to 395.00 ng/mL). The Wisconsin screening algorithm also incorporates provisions for when a single marker is elevated at a concentration greater than 10 standard deviations from the mean. One patient confirmed to have SV CAH was collected at 2 h of life. In this specimen, 3 markers were elevated, but both ratios were greater than 10 standard deviations from the mean. In addition, the Wisconsin modified algorithm, incorporating a 10 standard deviations cutoff from the mean, also detected a non-classical CAH patient. To address the potential for false negatives due to missed interpretations, the algorithm includes a “possible abnormal” reporting option to be used when four of the five markers are outside three standard deviations. The one-year prospective study achieved a 95% reduction in false positive rate to 0.04%, even with a conservative algorithm allowing for a “possible” abnormal interpretation. This tempered approach may be an appropriate alternative to minimize false negatives, but not substantially increase the false positive results. During the prospective study, 5 CAH patients were identified out of 63,725 analyzed (incidence of 1 in 12,600). This is higher than the reported incidence of 1 in 20,000, and suggests that it is likely no cases of CAH were missed. 

The impact of the second-tier test on the entire newborn screening program is significant. Studies have demonstrated that the second-tier test for CAH reduces the burden of follow-up care and has improved clinical practice by minimizing the need for sub-specialty, endocrine evaluations [34]. In addition, the second-tier test performed on the initial specimen is cost-effective—when compared to the alternative of additional testing, either repeat newborn screens or confirmatory tests [27]. Lastly, abnormal CAH screening results that were once considered to be “most likely a false-positive” by physicians are now likely to be a true case. This places a higher priority on the need to obtain endocrine consult and appropriate confirmatory testing in a timely manner. Changes in laboratory practices, such as implementation of second-tier testing, should be accompanied by proper notifications and education for care providers.

The risk of missed CAH cases, whether after first- or second-tier analysis, will always remain a concern for programs. The decrease in flux through the steroid synthesis pathways, largely due to cortisol inhibition or the lack of mature enzyme function, will continue to contribute to false negative rates. The incorporation of a more complete steroid panel, including measuring steroids within the mineralocorticoid pathway, may allow for a more thorough assessment of the pathway. Additionally, clinically relevant factors—such as birth weight or the timing of collection—should be incorporated into all screening algorithms, similar to what has been proposed by Rinaldo et al. [35]. In the past, NBS programs have been somewhat hampered by limitations of statistical assessments and the ability to account for multiple factors. It is likely that molecular analysis would also increase specificity [36]. However, not all mutations can be reliably detected in the screening setting and therefore may ultimately limit the sensitivity [37]. Furthermore, even with the incorporation of molecular analysis, a first-tier biochemical assay needs to be performed, and at this time, the LC–MSMS second-tier method, with a relatively long run time, cannot replace the immunoassay. Our future studies will move toward a more thorough examination of the steroid synthesis pathway, assessing the weighted impact of other analytes and physiological factors, to guide disease identification.

This study replicates and expands upon previous work to validate and implement a second-tier assay for classical CAH. Multiple steroids and ratios, along with appropriate adjustment for physiological factors, collection time and birth weight, enabled Wisconsin to suggest a more comprehensive screening algorithm that has not been previously delineated.

## Figures and Tables

**Table 1:** Parameters for detection of steroids and internal standards. Table 1 provides the multiple reaction monitoring (MRM) transitions, retention times, and collision energy for each steroid and its corresponding internal standard. Q = quantifying transition, I = identifying transition, MRM = multiple reaction monitoring.

Steroids and Internal Standards	MRM Transitions	Retention Time (min)	Collision Energy (V)
17-hydroxyprogesterone (Q)	331.2→97	3.1	60
17-hydroxyprogesterone (I)	331.2→109		60
Cortisol (Q)	363.3→121.2	2.25	30
Cortisol (I)	363.3→327.1		24
Androstenedione (Q)	287.2→97	2.9	28
Androstenedione (I)	287.2→109		31
21-Deoxycortisol (Q)	347.3→311.2	2.5	25
21-Deoxycortisol (I)	347.3→109.01		46
D8-17-hydroxygrogesterone	339.2→113.1	3.1	47
D4-Cortisol	367.1→121.1	2.25	34
D7-Androstenedione	294.2→100.1	2.9	27
D8 21-Deoxycortisol	355.2→319.2	2.5	25

**Table 2:** Injection Profile. Table 2 provides the liquid chromatography injection profile for separation of the steroids. %B = percentage of mobile phase B at the corresponding time.

Time (min)	%B	Flow Rate (mL/min)
0.01	42.5	0.25
4.4	100	0.25
4.45	100	0.25
4.5	100	0.5
6.5	100	0.5
6.55	100	0.25
6.6	100	0.25
6.61	42.5	0.25
8.7	42.5	0.25

**Table 3:** Distribution of steroid concentrations within the normal population. Table 3 provides the concentrations for each steroid (17OHP, 4-A, 21) and both ratios within the normal population. The median, mean, 3 standard deviation (3stdev), and 99th percentile (0.99) values are provided. The normal population is stratified by birth weight and time of collection. In situations where there was no statistical difference between the stratified groups, the data is combined. BW = birth weight.

		17-Hydroxyprogesterone (ng/mL)		4-Androstenedione (ng/mL)		(17-OHP + 4-A)/F)	(17-OHP + 21)/F)	21-Deoxycortisol (ng/mL)
BW (g)		24–168 h	<24 h	24–168 h	<24 h	No Stratification by Timing	No Stratification by Timing	No Stratification by Timing or BW
≤1500	count	103	106	103	106	209	209	783
	median	3.31	4.55	0.88	1.84	0.26	0.19	0.15
	mean	5.05	6.16	1.20	2.52	0.74	0.57	0.21
	3stdev	18.72	20.99	4.56	8.64	5.43	4.52	0.78
	0.99	18.75	25.31	5.83	8.60	6.13	4.44	0.92
1501–2499	count	121	99	121	99	220	220	
	median	1.07	2.79	0.81	1.62	0.15	0.10	
	mean	1.65	3.18	1.07	2.07	0.41	0.28	
	3stdev	6.26	9.05	3.80	7.92	2.59	1.86	
	0.99	6.91	9.12	4.16	12.25	2.45	1.63	
≥2500	count	256	98	256	98	354	354	
	median	0.81	1.01	0.62	0.98	0.08	0.05	
	mean	0.89	1.56	0.71	1.42	0.17	0.12	
	3stdev	2.30	6.26	1.92	5.98	0.85	0.62	
	0.99	2.50	6.98	2.02	6.45	1.00	0.76	

**Table 4:** Summary of confirmed congenital adrenal hyperplasia (CAH) cases identified by newborn screening. Table 4 provides the concentration of 17OHP measured by the first-tier assay and the concentrations of 17OHP, 4-A, 21, and ratios measured by the second-tier assay within confirmed cases. The non-shaded cases were evaluated as part of the second-tier assay validation. The shaded cases were identified during the one-year prospective study. The form of CAH confirmed within each patient is provided. For some patients (labeled as only classical), the endocrinologist did not distinguish between salt wasting and simple virilizing forms of CAH. SW = salt wasting, SV = simple virilizing.

Patient #	17-OHP	Birthweight (g)	Age at Collection (h)	17-OHP	4-A	21	(17-OHP + 4-A)/F)	(17-OHP + 21)/F)	Form of CAH
	(ng/mL)			(ng/mL)	(ng/mL)	(ng/mL)			
	1st tier			2nd tier	2nd tier	2nd tier	2nd tier	2nd tier	
1	>189	3780	56	84.70	55.10	50.50	6.99	6.76	Classical-SW
2	116	3030	53	13.90	17.20	5.75	2.16	1.36	Classical-SV
3	>189	3062	24	60.10	8.97	38.60	12.16	17.38	Classical-SW
4	>220	4190	26	48.20	10.40	13.90	2.56	2.71	Classical-SW
5	125	3677	25	20.40	6.19	16.00	1.44	1.97	Classical
6	>220	3602	24	33.90	9.51	5.18	1.56	1.41	Classical-SW
7	>220	2840	27	71.10	38.70	11.60	20.26	15.26	Classical-SW
8	62	2860	24	7.23	3.00	1.63	0.99	0.86	Classical-SW
9	48	4079	25	6.14	2.63	0.83	1.08	0.86	Classical-SW
10	156	2730	45	14.10	2.87	3.66	1.17	1.22	Classical
11	72	4100	36	8.56	2.37	5.69	1.64	2.14	Classical
12	33	3340	24	3.02	1.65	4.98	0.32	0.56	Non-classical
13	425	2850	48	294.00	364.00	15.80	37.82	17.80	Classical-SW
14	365	3470	25	78.50	43.40	11.30	9.03	6.65	Classical
15	418	3157	37	395.00	20.60	68.10	28.66	31.94	Classical-SW
16	53	1180	2	19.10	2.68	1.14	34.57	32.13	Classical-SV
17	129	3610	45	19.90	4.45	22.00	1.25	2.15	Classical
18	96	3735	33	10.60	3.07	1.36	4.08	3.57	Classical-SV
19	>200	3530	24	359.00	76.60	38.90	28.10	25.67	Classical
20	>200	3055	45	135.00	275.00	2.15	31.06	10.39	Classical-SW
21	>200	2650	35	172.00	234.00	6.14	57.67	25.30	Classical
22	123	4100	63	21.30	14.60	7.48	3.55	2.85	Classical
23	>200	2970	27	74.10	64.60	23.10	8.46	5.93	Classical-SW
